# Chicago Medical Society

**Published:** 1882-08

**Authors:** 


### Society Reports.

ARTICLE V.

### CHICAGO MEDICAL SOCIETY.

Stated Meeting, July 17, 1882, Dr. J. H. Hollister, President, in the Chair.

### INSANITY—TETANUS.

After reading and approval of the minutes of the last meeting, Dr. C. T. Fenn presented a cranium, an inferior maxillary bone, and a rib from a man who died insane, and presented the following history:

Ashbel D. was born of Puritan ancestry, in Cornwall, Conn., about 1796. He died in Tallmadge, Ohio, in 1878, æt. about eighty-two. In his younger days he possessed a comely person, pleasing manners, and a fondness for society. He was a saddler and harness-maker, industrious and thrifty, especially neat in his dress, and of good morals. He never married. D. first came to Ohio on the early settlement of the Western Reserve, about 1823, working at his trade, but after a time he returned east as far as New York.

He loved and was rejected; an authentic incident of that period. It is known, also, that she who was the object of his affection was applied to by several men, but adhered to her early determination, and remained single. She is living, at the age of eighty-five.

A history of hereditary insanity was not ascertained. After his love disappointment, he took to drinking, and had delirium tremens, after which his health seemed impaired, and he took to traveling, and his mind wandered from that period.

When forty-six years old, he returned to Ohio. He now appeared an old man. His hair was thick and gray, his face wrinkled, and his keen black eyes wore a peculiar expression characteristic of the insane. His body was spare, of medium height, straight, lithe and active. He died in the same house whose hospitality he never lacked, but from which he often wandered to great distances. He made his journeys on foot, alone, refusing offers to ride, and not partaking of food. He endured fasts, and unfriendly heat and cold without complaint. His circuit was gradully fixed, covering three or four counties, in which he had perhaps as many rural haunts, finding in each a temporary home, unwelcome to any with whom he might claim kinship. His advent was not entirely unwelcome in the busy season, for he was inclined to be industrious and useful. He was fond of attending to fires, had a special aversion to sponging, and could not bear restraint, but would leave. He generally went off by night, without notice, and without preparation, returning after a few weeks or months, as the case might be, in the same surprising manner.

He was prone at twilight, or in darkness, to drum with the back of his right hand on tin pans held in his left, going some distance from the house to perform. His knuckles were constantly abraded on that account. His aim was to frighten away spirits who were after him or tortured little children. He had hallucinations of sight, as well as of hearing. His apparel was sometimes highly fantastic. Seemed never to have had any disease except what was alluded to above. Seemed to have been temperate subsequently to his attack of delirium tremens. He chewed excessively, but after awhile forgot the use of tobacco also.

During the last years of his life he became steadier and quieter, and could talk rationally for long periods at a time. It seems that he was a Christian, and at times he had been extremely profane. Dr. S. S. Wright, of Ohio, gives the following notes of the autopsy :

The vessels of the brain were all full of blood. There were slight adhesions of the pia mater between the convolutions, and a little pus on the surface of the cerebellum. A curious loop of blood-vessels extended along the floor of each of the lateral ventricles, plexus-like, and full of arterial blood, bound by delicate fascias. There was some softening of the brain, and a few brown spots on the vertex, like apoplectic marks. The size of the organ appeared normal. The stomach was softened and thickened, with about two or three ounces of muco-pus in it. There were evidences of congestion almost everywhere; in pericardium, lungs, peritoneum and intestines. Liver was very small and hard. Pancreas enlarged; spleen small and hard; kidneys normal. There was calcareous degeneration of the arteries.

Dr. Fenn remarked that Ashbel's skull appeared symmetrical and normal at first, but, on close inspection, it was found that the calvarium was quite thick, somewhat asymmetrical, and that the cranial sutures were all strongly ossified. Looking at the skull from above, there was a marked want of symmetry within it, the right side being fuller, and five-eighths inch larger in diameter than the opposite side. Looking at it externally, there was a marked flatness of the left side. Looking at the petrous portion of the temporal bone, the internal auditory foramen on the left side was half an inch nearer the median line than on the right side. The left jugular foramen was three times larger than the opposite one.

Dr. J. H. Hollister related the case of a murderer, who had never been insane, but a violent man. His skull was fully as heavy and thick as the one under consideration. Looking at the petrous portion of Ashbel's skull, the doctor remarked that there was a deposit which showed long-continued malnutrition, if not an inflammation, of the endosteum. He believed that was calcification, not real ossification, which was an extremely rare occurrence. In was claimed by some, even in our day, that insanity sometimes took place without accompanying pathological changes. There were other cases in which all sorts of pathological changes would take place, and yet the patient remain perfectly sane. And between these two limits there were an incredible variety of cases. The subject was a delicate one. There were persons in whom a certain behavior would be considered a sign of mental aberration, which in others would be considered proper. As to the inheritance of the disease, he believed that after a certain age was safely attained, there was no more hereditary tendency. He related the case of a man, a wonderful athlete, most muscular and sprightly, who fell from a rope and hit his head against a sidewalk. In a few weeks he became insane, and had to be kept in confinement for twenty-two years, dying at the age of seventy-one.

Dr. Fenn, in reply to Dr. Hollister, said that in the specimen in hand, the skeleton was remarkably light, with attenuation of bone. The pachionian bodies had almost worn through the skull, which was here no thicker than paper. The asymmetry of the skull was not necessarily an index of insanity. Said he: "I believe that classes of people by imitation will become insane, in the same way that epidemics of chorea occur." In times of political excitement people may be roused to murder, and that was an insanity of imitation.

A case had come under his observation, said Dr. W. H. Curtis, in which there was a deposit of bone $1 x \frac{3}{4} x \frac{1}{6}$ inches in dimensions in the meninges of the brain. Whether it was caused by the insanity, or caused the latter, he could not say.

Dr. Hollister said an author claimed habitual congestion of the brain from alcohol as one of the most common causes of insanity, but, in the doctor's mind, a peculiar physical organization was necessary for insanity to develop. This consisted of an emotional nature, a high nervous organization, and a debilitated physique. In these cases, stimulants, sexual excesses, political or religious excitements would induce insanity. One present danger to the race was an excessive mental education at the expense of the physical organization. He had known of a family of five who were all insane in some way, though the father and mother possessed high mental refinement. He believed the progeny of several generations of people who had specially exercised their minds was liable to prove mentally debilitated, and this would perhaps apply to the royal families of Europe. In his experience, he had found that the business men of Chicago especially suffered from headache, and some had to leave the board of trade and take a vacation to relieve their minds. The doctor remarked that there was one day a week consecrated to mental rest, but that two days would be still better. He thought we would have soon to return to the Roman system of physical education, specially in institutions for girls, for the crop of insanity in New England was fearful. He said a tendency to insanity should deter one from entering a profession.

### TETANUS.

Dr. D. I. Scheppers made the statement that out of five cases of shot wounds following the fourth of July this year, he had had one case of tetanus; that the year previous he did not have one case of tetanus out of fifty cases of wounds; he wished to know the opinion of members in regard to the causes of prevalence of tetanus.

Dr. Kiernan said that the pathological results found in patients dead of tetanus were generally secondary, and that there was no invariable pathological change. He had met spurious cases where the mind of the patient was the only factor. There were regions, as Japan, Corea and Long Island, where the disease was of frequent occurrence. He had seen a case of tetanus in the new-born. In that case, the mother had been seduced, and afterward chased from place to place, and when the child was born, the least noise would throw it into spasms. Marked changes in the pachionian bodies had sometimes been found.

Dr. H. D. Valin referred to notes of epidemic tetanus in the translations of the June number of the JOURNAL, and mentioned the case of a patient in the County Hospital who had recovered from tetanus after stretching of the nerves.

Dr. Kiernan recommended the further use of conium in tetanus, and Dr. Hollister made the same suggestion, as that medicine had given him good results in infantile convulsions. He used the tincture, but Dr. Kiernan thought the tincture of conium was inert, and recommended the fluid extract.

